# A vision of 14 T MR for fundamental and clinical science

**DOI:** 10.1007/s10334-023-01081-3

**Published:** 2023-04-10

**Authors:** Steve Bates, Serge O. Dumoulin, Paul J. M. Folkers, Elia Formisano, Rainer Goebel, Aidin Haghnejad, Rick C. Helmich, Dennis Klomp, Anja G. van der Kolk, Yi Li, Aart Nederveen, David G. Norris, Natalia Petridou, Stefan Roell, Tom W. J. Scheenen, Menno M. Schoonheim, Ingmar Voogt, Andrew Webb

**Affiliations:** 1 Tesla Engineering Ltd., Water Lane, Storrington, West Sussex, RH20 3EA UK; 2 Spinoza Centre for Neuroimaging, Amsterdam, The Netherlands; 3 Computational Cognitive Neuroscience and Neuroimaging, Netherlands Institute for Neuroscience, Amsterdam, The Netherlands; 4 Experimental and Applied Psychology, Vrije University Amsterdam, Amsterdam, The Netherlands; 5 Experimental Psychology, Utrecht University, Utrecht, The Netherlands; 6 Philips, Best, The Netherlands; 7 Department of Cognitive Neuroscience, Maastricht University, Maastricht, The Netherlands; 8 Maastricht Brain Imaging Centre (MBIC), Maastricht University, Maastricht, The Netherlands; 9 Wavetronica, Padualaan 8, 3584 CH Utrecht, The Netherlands; 10 Donders Institute for Brain, Cognition and Behaviour, Centre for Cognitive Neuroimaging, Radboud University, Nijmegen, The Netherlands; 11 Department of Neurology, Center of Expertise for Parkinson and Movement Disorders, Donders Institute for Brain, Cognition and Behaviour, Radboud University Medical Centre, Nijmegen, The Netherlands; 12 Radiology Department, Center for Image Sciences, University Medical Center Utrecht, Utrecht, The Netherlands; 13 Department of Medical Imaging, Radboud University Medical Center, Nijmegen, The Netherlands; 14 Independent Researcher, Magdeburg, Germany; 15 Department of Radiology and Nuclear Medicine, Amsterdam University Medical Centers, Amsterdam Cardiovascular Sciences, University of Amsterdam, Amsterdam, The Netherlands; 16 Erwin L. Hahn Institute for Magnetic Resonance Imaging UNESCO World Cultural Heritage Zollverein, Kokereiallee 7, Building C84, 45141 Essen, Germany; 17 Department of Clinical Neurophysiology (CNPH), Faculty Science and Technology, University of Twente, Enschede, The Netherlands; 18 Neoscan Solutions GmbH, Joseph-von-Fraunhofer-Str. 6, 39106 Magdeburg, Germany; 19 Department of Anatomy and Neurosciences, MS Center Amsterdam, Amsterdam Neuroscience, Amsterdam UMC, Vrije Universiteit Amsterdam, Location VUmc, P.O. Box 7057, 1007 MB Amsterdam, The Netherlands; 20 Department of Radiology, C.J. Gorter MRI Centre, Leiden University Medical Center, Albinusdreef 2, 2333 ZA Leiden, The Netherlands

**Keywords:** Ultra-High Field MRI, Neuroimaging, Neuroscience, Brain disorders, Medical applications

## Abstract

**Objective:**

We outline our vision for a 14 Tesla MR system. This comprises a novel whole-body magnet design utilizing high temperature superconductor; a console and associated electronic equipment; an optimized radiofrequency coil setup for proton measurement in the brain, which also has a local shim capability; and a high-performance gradient set.

**Research fields:**

The 14 Tesla system can be considered a ‘mesocope’: a device capable of measuring on biologically relevant scales. In neuroscience the increased spatial resolution will anatomically resolve all layers of the cortex, cerebellum, subcortical structures, and inner nuclei. Spectroscopic imaging will simultaneously measure excitatory and inhibitory activity, characterizing the excitation/inhibition balance of neural circuits. In medical research (including brain disorders) we will visualize fine-grained patterns of structural abnormalities and relate these changes to functional and molecular changes. The significantly increased spectral resolution will make it possible to detect (dynamic changes in) individual metabolites associated with pathological pathways including molecular interactions and dynamic disease processes.

**Conclusions:**

The 14 Tesla system will offer new perspectives in neuroscience and fundamental research. We anticipate that this initiative will usher in a new era of ultra-high-field MR.

## Introduction

MRI is a key technology in both neuroscience, and medicine. In the Netherlands an open consortium has been formed consisting of the academic parties: Amsterdam Medical Centre; Leiden University Medical Centre; Maastricht University; Radboud University; Radboud University Medical Centre; Spinoza Centre for Neuroimaging Amsterdam; University Medical Centre Utrecht to form the Dutch national 14 Tesla MRI initiative in medical sciences (DYNAMIC in short). This paper describes the DYNAMIC’s approach for establishing a 14 T MR system.

**Fig. 1:**
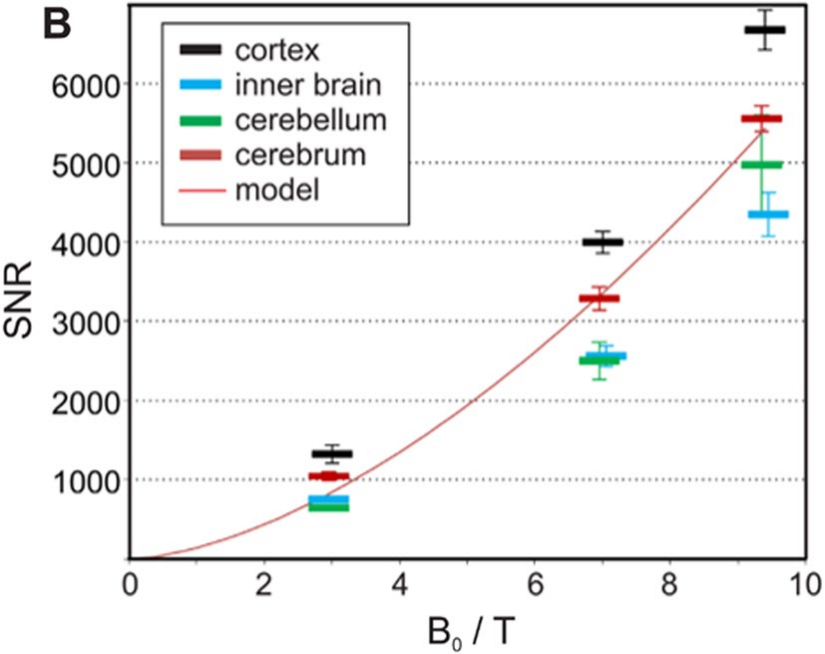
Signal-to-noise (SNR) values measured in four different brain compartments as a function of static magnetic field strength (*B*_0_). The red line represents fitting results on the SNR over the entire cerebrum as SNR proportional to *B*_0_^1.65^. Taken from [1]

The primary motivation for moving to 14 T is sensitivity. In MRI the signal intensity is proportional to the voxel volume and hence improvements in spatial resolution that access different biologically relevant spatial domains require large increases in sensitivity to achieve the necessary gain in resolution. As shown in Fig. 1, the signal-to-noise ratio in the human head increases with *B*_0_^1.65^ [1] implying that a doubling in field strength will lead to a factor 3 increase in sensitivity, which is the gain we anticipate compared to the current ultra-high-field standard of 7 T. In the centre of phantoms, a higher power dependency has been found of *B*_0_^1.94^ [2], but this is unlikely to be realised in vivo.

Historically, the MRI-community has moved from 1.5 T (1980s) to 3 T (1990s) to 7 T (early 2000s), all of which are now FDA approved clinical devices. Progress beyond 7 T has been limited to a number of systems working at 9.4 T (Maastricht and Tübingen), 10.5 T (Minnesota) and two 11.7 T systems that are currently coming into service (Neurospin, Paris; NIH Bethesda). Progress beyond 7 T has been hampered by limitations in the superconducting magnet technology employed. Almost all current magnets use niobium titanium (NbTi) as the superconducting material, which has a critical field (above which it will not function) of about 12 T [3]. This limitation has prevented a large step in static magnetic field strength and imposed significant design constraints on systems operating close to this limit. To move beyond 12 T it is necessary to take the step to a different magnet technology. After a lengthy consultation process DYNAMIC has selected a magnet based on high temperature superconductor (HTS) technology, which in the case of the superconductor chosen has a critical field of 28 T. The proposed 14 T system is in the mid-range field of this superconductor, resulting in an efficient and compact magnet design.

The high expense of the proposed system and the likely similar high cost of future systems, means that it is unlikely that a large number of such systems will be built in the medium term, and in the long term it is unlikely that they will be used in clinical routine. As a consequence, DYNAMIC aims to answer fundamental questions in medicine and neuroscience rather than develop techniques for routine diagnosis. This article describes in general terms how DYNAMIC will target such fundamental breakthroughs in a pragmatic and structured fashion. The brain is more accessible to MRI than organs in the torso and moves to higher static field strength have for this reason initially focused on the brain. We shall follow the strategy of establishing a robust setup for brain imaging in parallel to developing techniques for other organs. This gives three domains of research, where significant progress can be expected:

1. *Neuroscience*. Where we will be able to study the brain with both structural and functional imaging at the spatial scale of its fundamental processing units, the cortical layers, and columns.
2. *Brain disorders*. Where we shall be able to study the dynamic processes associated with brain disorders at the molecular level.
3. *Medicine*. By interrogating the micro-environment of diseased regions and their response to treatment.

For each of these three domains a group of experts within the field have consulted broadly within the Dutch scientific community to produce an over-arching narrative for each domain. In this review article we shall describe our initial methodological approach, the hardware configuration that we have selected and the broad scientific goals that we have set ourselves.

## Methodology and hardware configuration

There are well known challenges associated with radio-frequency transmission and power deposition (specific absorption rate, SAR) associated with a move to higher field strengths, which have direct implications for the choice of MR acquisition techniques [3–6]. With hindsight it now appears that the major step up in technology was from 3 to 7 T, because in broad terms, this was the first time that the MRI community had to deal with wavelengths of the radio-frequency field that were considerably shorter than the spatial extent of the object being investigated. Initial experience from moving to 10.5 T has indicated that the solutions already developed for dealing with short wavelengths at 7 T, will also work well at higher field strengths [7, 8]. It also seems that the SAR problem is less severe at ultra-high-field strengths than originally thought [9–11], although care must be taken that inhomogeneities in the transmit field do not lead to hotspots with high SAR [12].

The expectation is that an upscaling of the 7 T technology to 14 T will give a viable imaging system provided that only relatively low flip angles are used. This is not to underestimate the challenges associated with imaging at such high static magnetic field strengths, but rather to develop a scientific strategy that maximizes the potential gains from such a system while also catering adequately for novel techniques. We initially intend to establish a robust proton imaging and spectroscopy setup for measurement in the brain using acquisition techniques that do not require spin-echoes and can hence use low excitation angles up to the Ernst angle. In parallel we shall develop methodologies for imaging outside the brain, and for using X-nuclei. In this way, we will be able to make breakthroughs in neuroimaging utilizing the robust setup while developing and exploring exciting new technologies, which in turn will lead to breakthroughs in all domains.

For brain imaging we will use a standardized experimental setup designed to ensure near optimal and reproducible conditions. The combination of imaging readouts that use low excitation angles, combined with inversion pulses, will allow us to cover a broad range of experiments that are of considerable importance for examining the brain and its function. With such experiments we intend to avoid constraints imposed by SAR, but changes in relaxation times also affect the design of imaging protocols and the results attainable. For the brain we can use results obtained at lower field strengths to predict the relaxation times that we will encounter. These are given in Table 1, alongside values at 7 Tesla for reference.

**Table 1:** Variation of relaxation times in the brain with static field strength

Static field (*T*)	Grey matter			White matter			Blood	
	*T*_1_ (s)	*T*_2_ (ms)	*T*_2_* (ms)	*T*_1_ (s)	*T*_2_ (ms)	*T*_2_* (ms)	*T*_1_ (s)	*T*_2_ venous (ms)
7	1.8	49	28	1.2	57	27	2.1	20
14	2.3	34	16	1.6	38	15	3	6

The values are calculated/measured according to the following literature: grey and white matter *T*_1_ [118]; Grey and white matter *T*_2_ [119]; Grey matter *T*_2_* [120]; White matter *T*_2_* [121]; Blood *T*_1_ [122]; Venous blood *T*_2_ [123]

As we shall initially eschew spin-echo-type experiments the shorter *T*_2_ values are not relevant, but the reduced *T*_2_* will limit the readout duration of EPI experiments, and the short *T*_2_ of venous blood will make this invisible in all but short TE experiments.

With 2D EPI and simultaneous multi-slice acquisition we will be able to obtain images with high temporal but restricted spatial resolution. For high spatial resolution we shall utilise a segmented 3D EPI experiment [13–15] potentially in combination with high density receiver arrays and CAIPIRINHA [15–17]. We shall also explore the potential of the recently introduced Echo-Planar Time-Resolved Imaging (EPTI) [18] technique for quantitative imaging. The use of inversion recovery sequences will allow the anatomical imaging experiments of MPRAGE, and MP2RAGE [19], as well as for measuring cerebral blood volume (CBV) using VASO [20], and arterial spin labelling (ASL) based on FAIR [21], which can offer increased sensitivity albeit with limited volume coverage. In addition to the conventional imaging experiments, we see considerable potential in immediately implementing spectroscopic imaging (SI) of protons. In parallel, the technology for measuring X-nuclei and protons outside the brain will be developed, including multi-channel RF transmit coils and high-density receiver arrays.

### Magnet

The most important element of the system is undoubtedly the magnet. Following a lengthy consultation and evaluation process a high temperature superconducting (HTS) magnet was selected [22] to be manufactured by Neoscan Solutions GmbH (Magdeburg, DE). In [22] it was proposed to use a first generation HTS wire, the ceramic (and rare-earth-free) superconducting compound consisting of bismuth strontium calcium copper oxide (BSCCO, pronounced ‘bisko’) embedded within a silver-containing matrix, which has a critical field of 28 T. Stable magnets of 25 T have previously been constructed using BSCCO tape [23]. As compared to a magnet based on a low temperature superconductor (LTS), an HTS magnet has the following generic advantages:

1. HTS wire has a high current-carrying capacity, enabling a compact and relatively light magnet design.
2. The compact design means that the stray field volume is similar to that of an unshielded 7 T system
3. High critical temperature safety margin of 16 K means that temperature monitors within the magnet can shut off gradients and prevent damage caused by gradient magnet interactions.
4. The magnet can be conductively cooled: a dry magnet is less complex with no need for a liquid helium dewar, it is safer as the cryostat is not a high-pressure vessel and there is no need for a helium quench pipe, and no refilling of helium.
5. In the event of a quench a high percentage of the superconducting tape is reusable. This drastically shortens the repair time compared to an LTS-based magnet.
6. An uncast magnet construction is easy to repair.

There are also some disadvantages to using HTS. A technical disadvantage is that it is not possible to construct HTS joints, so magnets run in driven rather than persistent mode. With the high stability of current power supplies this does not lead to a penalty in terms of field stability. For the proposed design the additional cooling power required to cover ohmic losses is 1 W at 4 K, which is easily achieved. The major disadvantage is probably the higher price of such a system. A detailed description of the magnet design is to be found in [22]. For reference a comparison of the salient characteristics with those of some existing LTS magnets is given in Table 2.

**Table 2:** Comparison of various passive shielded UHF magnets for whole-body MRI

Type	7 T 900	9.4 T 820	9.4 T 900	11.7 T 580	14 T 820
Magnet material	LTS NbTi^a^	LTS NbTi	LTS NbTi	LTS NbTi	HTS BSCCO
Cooling approach	LHe bathed	LHe bathed	LHe bathed	LHe bathed	Cryogen-free
Shielding pattern	Passive	Passive	Passive	Passive	Passive
Warm bore diameter	900 mm	820 mm	900 mm	680 mm	820 mm
Width	2.38 m	2.56 m	2.96 m	2.73 m	1.4 m
Length	3.37 m	3.70 m	3.72 m	3.66 m	2.6 m
Mass	32 tonnes	47 tonnes	57 tonnes	60 tonnes	16 tonnes
DSV with 5 ppm homogeneity^b^	45 cm	40 cm	45 cm	30 cm	> 45 cm
5 Gauss contour (radial × axial)	17.8 m × 22.5 m	20.5 m × 25.9 m	21.8 m × 27.6 m	21.4 m × 27.0 m	17.0 m × 21.5 m
Stored energy	79 MJ	152 MJ	182 MJ	194 MJ	148 MJ
Size comparison					

Except for the type of 14 T 820, parameters of other magnets are referred to [124]

^a^Abbreviations in the table: DSV, diameter of a spherical volume; LHe, liquid Helium; NbTi, Niobium Titanium alloy; BSCCO, Bismuth strontium calcium copper oxide superconductor

^b^Homogeneity in peak to peak

### Radiofrequency proton resonator for the head

Wavetronica BV (Utrecht, NL) is the company which will construct the proton head resonator in the initial phase of the project. Development will follow a two-step process to initially build an 8Tx, 32 Rx coil (including preamps) to be followed by a 16Tx 64Rx coil. This will incorporate a 32 channel *B*_0_ shim array (including amplifiers) which will be implemented as a separate layer on top of the receive array.

Based on insights from RF coil development at 7 T, 10.5 T and initial simulation studies at 14 T [24], it is known that the RF coils for 14 T MRI systems will look different from current clinical RF coils. Arrays of multiple RF transmit coils can steer the EM waves towards the imaging target that together with magnetic field gradients can create a homogeneous flip angle distribution in the region of interest [10]. Based on work performed at 7 and 9.4 Tesla, accounting for the shorter wavelength at 14 T, arrays containing high numbers of transmit channels and arrays integrating dipole antennas in combination with loop coils, would appear to be the best candidates in terms of transmit and SAR efficiency [25–27]. The exact role of the RF shield, and the form of the dipole array in terms of folded versus straight dipoles will require extensive simulations to come up with the optimum sensitivity. The addition of high permittivity material, as recently demonstrated at 10.5 T, is also an interesting method to increase the sensitivity of high-field arrays [28].

Parallel transmit methods such as *kT*-points, universal pulses and sequences optimized to lower peak SAR can further increase the RF coil performance [29]. Sufficient common mode rejection and the use of low loss cabling can ensure high RF power delivery by transmitter elements that are mounted inside a rigid housing. Further away from the subject, a *B*_0_ shim array can be incorporated, acting partially as a RF shield for the transmitter array and functioning mainly to counter any *B*_0_ disturbances that will arise due to intra-subject magnetic susceptibility deviations. A *B*_0_ shim array will be integrated in the RF coil setup [30–32] and become part of the overall *B*_0_ shimming system, with coil currents determined by the procedure described in [30]. Previous simulation studies suggested that at 14 T, the diameter of the RF shield will have a strong impact on the SNR and, therefore, becomes part of the optimization strategy [33]. Moving closer to the subject, a high density receive array as close to the subject as possible that accounts for subject shape and dimension variation, can be realized with a (partially) flexible receive array [34]. This way, tissue loss remains dominant for receiver arrays beyond 128 elements and by integrating low noise preamps in the elements, RF coupling between them can be managed to maintain high sensitivity.

### Gradient system and console

Tesla Engineering Ltd. (West Sussex, UK) will construct the gradient system. The HFC45 (Tesla Engineering Ltd. model designation) gradient coil envisaged for the 14 T system has some high-end features, such as peak strength of 120 mT/m and slew rate of more than 250 T/m/s. While the performance levels of HFC45 may seem unremarkable, it should be noted these parameters are achieved with single gradient drive amplifiers per axis, with 1200 A and 1350 V peak current and voltage. In addition, the coil thickness is only 100 mm, limited by the magnet warm bore, which is less than half that of coils used in the highest end systems (for example the GE Magnus gradient coil is 235 mm thick). With a gradient bore of 60 cm, it will be possible to carry out whole body imaging, rather than limit the system to head only. The HFC45 design employs direct-cooled *X*, *Y*, and *Z* coils to promote very high steady stage gradient values, *G*_rms_, of over 37 mT/m in all three axes simultaneously. One of the major challenges will be containing the Lorentz forces at 14 T, which will be double those of a 7 T system. Magnet/gradient interaction is of particular interest, ultra-low eddy currents, mechanical de-coupling, and low acoustic noise (force balanced), will be key targets of the development process. It will be necessary to utilize high strength and directional reinforcement within the gradient coil to maximize stiffness and toughness, within the confines of the mechanical envelope. The coil will incorporate second- and third-order shims (*Z*^2^, *ZX*, *ZY*, *X*^2^–*Y*^2^, *XY*, *Z*^3^, *Z*^2^*X*, *Z*^2^*Y*, *Z*(*X*^2^–*Y*^2^), *ZXY*, *X*^3^, *Y*^3^) driven at 10A.

The system will be controlled by a fully digital console architecture supplied by Philips Medical Systems. This will be capable of driving 32 channel Tx, 128 channel Rx and includes the RF amplifiers (32 × 1 kW) for 16 channel transmission at the proton frequency and the other 16 combined as 4 × 4 kW for 4 channel broadband transmission. Philips will take responsibility for system integration support.

## Research fields

### Neuroscience

Over the past three decades, MRI has revolutionized non-invasive neuroscience, and has offered unprecedented advances in our understanding of the structure and function of the human brain.

Recent developments in hardware technologies, data acquisition and analysis methods have boosted our capability for detailed and rapid non-invasive brain imaging. Neuroscientists are now equipped with tools for addressing questions on brain structure, function, and their relationship with ever-increasing sophistication. For instance, using sub-millimeter fMRI at ultra-high-field (≥ 7 Tesla) researchers have begun to map the neural substrate of human perception at spatial resolutions approaching the laminar and columnar levels [35–39]. Reaching such a mesoscopic level of resolution would be crucially relevant for revealing the anatomical substrate and the neuronal implementation of fundamental brain mechanisms, such as feedforward and feedback processing as well as the nature of cortical representations, which are at the basis of human cognition [40–42]. These mechanisms are also core components of influential computational models of brain processing (e.g., deep convolutional networks, predictive coding, Dynamic Causal Modelling). However, despite these advances, the current quality of empirical data is not sufficient to inform realistic computational models of neural processing and disambiguate the complexity of neuronal processes at the circuit level [39].

The increase in spatial resolution at 14 T may enable neuroscientists to *anatomically* resolve all layers of the cortex, cerebellum, subcortical structures and inner nuclei and their connectivity. This is crucially important to define anatomical brain regions *in-vivo*, allowing characterization of individual variability and correlations with cognitive and impaired function. Imaging of myelin-related signals at resolutions on the order of 0.2 mm or less can offer a view to the cortical laminar organization and small structures in subcortex [37, 43], Myelin protects axons and accelerates signal transmission. White matter is especially myelin dense, as it mainly consists of axons connecting neurons to each other, but grey matter also contains myelin. More myelinated grey matter structures in the brain tend to be those requiring raw processing speed, such as the primary motor, visual and auditory cortices. The myelin distribution has been used since the early twentieth century to map the human brain [44]. Studying the large-scale myeloarchitecture of the brain is possible in-vivo, using the *T*1-contrast in MRI, in some cases combined with iron-sensitive *T*_2_* mapping. At 7 T, this has already yielded impressive results in both cortical brain regions [45, 46] and the subcortex [43] at spatial resolutions on the order of 0.5 mm isotropic. Currently, spatial resolutions of at best approximately 0.3 mm can be achieved [47]. Unfortunately, many of the myelinated structures known from histology are too small to be observed by MRI at this resolution. Further improving this resolution to 0.2 mm and below will be an important step to visualize intra-cortical myelin differences and important structures in the cerebellum [48]. In addition to in vivo anatomical imaging, all ex vivo anatomical applications as well will benefit from 14 T high spatial resolution. For example, ex vivo diffusion MRI (dMRI) at 500 μm isotropic resolution and far below may become an important research tool for neuroanatomical investigations and for validation of in vivo diffusion MRI techniques.

The spatial resolution at 14 T may also enable to *functionally* resolve all layers of the cortex and investigate the inter-laminar interaction between brain regions, giving access to feedforward and feedback neural processing. The increase in spatial resolution at 14 T may also enable to functionally resolve all layers of the cortex and investigate the inter-laminar interaction between brain regions, giving access to feedforward and feedback neural processing. Humans construct predictive models of themselves and their environment, allowing them to make sense of incoming data. In line with this notion, the brain has been described as a ‘prediction machine’ that attempts to match incoming sensory inputs with top-down expectations [49]. Predictions are fed backward in the hierarchy and reciprocated by prediction error in the forward direction, acting to modify the representation of the outside world at increasing levels of abstraction, and so to optimize the nature of perception over a series of iterations. These computations occur at the level of the microcircuitry in which different layers have specific roles [40]. Ultra-high-resolution fMRI may allow to study communication at the level of resolution required to validate or refute such models. At existing magnetic field strengths, it has been feasible to map columnar (i.e., common to all layers in the same location) and laminar neuronal processes at the level of supra-, infra- and granular-layer compartments (laminar fMRI) (see reviews [37, 43]). Albeit remarkable, such achieved resolution is not yet sufficient to validate or refute models of neuronal communication and information transfers and to compare investigations in humans and animals. At 14 T, the expectation is that there will be sufficient spatial resolution (below 0.5 mm) and sensitivity to resolve neuronal responses in all six histological layers of the iso cortex. The two main approaches used at 7 T for obtaining laminar and columnar fMRI data, are gradient echo (GE) EPI and Vascular–Space–Occupancy (VASO) [20, 50], which can both be used at 14 T. With GE-EPI high resolution maps of the vasculature can be imaged and used to inform realistic models of the BOLD response and obtain truer laminar profiles. With VASO layer-dependent neural activity can be acquired with an effective resolution of 200 μm, pushing the boundaries of the current state-of-the art. The same resolution as for laminar fMRI is also needed to resolve feature-specific cortical columns, such as orientation columns in primary visual cortex. With the expected spatial resolution of 14 T it will become possible to discover functional columns, and thereby the coding principles, in mid- and higher level cortical areas. Importantly, combining anatomical information with functional measures at such a level of detail will make it possible to study fine-grained structure–function relationships and connectivity in individuals, and test theories and realistic computational models of cognition.

In parallel to these developments in anatomical and functional imaging, 14 T may open new frontiers in understanding brain metabolism and its relation to cognition. Spectroscopic imaging at 14 T may enable us to measure excitatory and inhibitory metabolic activity simultaneously, and thus to characterize the implementation of cognitive functions in terms of the dynamic excitation/inhibition balance of neural circuits. Current approaches to quantifying the excitation/inhibition balance rely on extracellular identification of excitatory or inhibitory neurons in well-defined circuits, or intracellular recordings, preventing in-vivo non-invasive application (e.g., [51, 52]). For example, it may be possible to directly measure GABA and glutamate concentrations in anatomically homogeneous cortical volumes and in a relatively short time, and thus to index changes in excitation/inhibition balance as a function of a participant’s cognitive state (e.g., during learning). The expected increase in spectral resolution and sensitivity at 14 T will both be crucial for improved detectability of these metabolites, particularly GABA, enabling measurements on the order of ~ 0.3 cc in few minutes (as compared to much larger volumes possible at 7 T, [53]). The increased chemical shift dispersion will mean that it may no longer be necessary to use editing experiments to identify GABA, and a short TE could be used to further increase sensitivity. The increased sensitivity to magnetic field inhomogeneities can in part be overcome by increasing the spatial resolution of MRSI methodology. This will make similar imaging performance possible for GABA as that for Glx at 7 Tesla [54]. 14 T MRI may provide the opportunity to measure glucose metabolism, including active metabolic pathways, at an unprecedented spatial resolution in the human brain (e.g., with deuterium ^2^H and carbon-13 ^13^C spectroscopy). DMI especially can be relatively easily translated to 14 T because of a homogeneous RF field due to the low resonance frequency of 2H (91 MHz at 14 T). Since spectral resolution will increase at 14 T, it may even be possible to separate glutamine and glutamate with DMI, enabling measurement of neurotransmitter cycling. Next to understanding healthy brain metabolism, measuring glucose metabolism with great precision is clinically relevant to detect early indicators of functional decline during aging.

In summary, in Neuroscience, 14 T MR may give the possibility of entering a number of biologically separate spatial domains in the living human brain, which have only been accessible up to now in post-mortem samples or animal models. This will enable an integral view that links neuronal organization, activity, and metabolism, which is critically needed to advance our understanding of human brain function.

### Brain disorders

Brain disorders are a major source of suffering and disability worldwide. In 2017, the total number of disability-adjusted life-years (DALYs) attributable to neurological disorders in the EU was 21 million, with a total number of deaths of 1.1 million; as a result, neurological disorders ranked third after cardiovascular diseases and cancers, representing 13.3% of total DALYs and 19.5% of total deaths [55]. In the upcoming years, these figures are expected to increase even more, in part due to an aging society [56].

Understanding the pathophysiology of brain disorders is essential to developing more efficient prevention and treatment strategies that may be able to contain or even reverse this concerning prospect. However, we are restricted to postmortem tissue and the occasional in vivo acquired biopsy sample as primary sources to elucidate how these diseases develop and progress. While postmortem tissue pathology may be the cornerstone for final diagnosis and to establish disease pathophysiology in the clinic, from an etiological standpoint these investigations are biased towards the end stage of progressive brain disorders. In addition, ex vivo studies on human tissue represent a static point in time instead of providing insight into the dynamics inherent to disease pathophysiology and are limited by small samples that can be studied under a microscope without considering the interaction between diseased tissue and the surrounding healthy brain. Finally, cell cultures and animal models—while enabling in vitro/vivo assessment of the molecular dynamics underlying brain disease pathology—often do not overlap enough with the in vivo human condition to provide sufficient data that can be used to treat or even prevent brain disorders.

To overcome these limitations, we need techniques that enable in vivo and preferably non- or minimally invasive analysis of the (dynamic) disease process, from its early development until the final stage, on a near-molecular level. MRI has been used to probe the structural and metabolic signature of diseases in vivo for quite some time using for instance MR spectroscopy and high-resolution structural imaging, however, with limited results mainly due to the low achievable spatial and spectral resolution. The introduction of ultra-high-field MRI (like 7 T) showed the advantages of the increased SNR of ultra-high-field systems [57] in diagnosing neurological diseases, such as epilepsy and MS; however, its specificity and dynamic range is still nowhere near that of in vitro cell-based or postmortem studies. 14 T MRI may provide a way to move forward by enabling assessment of the dynamic processes associated with brain disorders on a molecular level in vivo in humans. The significantly increased SNR at 14 T could enable visualization of fine-grained (high spatial resolution) patterns of structural brain abnormalities similar to microscopy that can be related to functional changes on a molecular level. In addition, dynamic (high temporal resolution) disease processes including their pathological pathways and associated interacting molecules (increased spectral dispersion) might be detected and measured, as well as molecular players not previously visualized in brain disorders, for example by measuring X-nuclei. All of this can be done in vivo in the living human brain, thereby providing not only a unique insight into brain disorders themselves, but also the interaction with their surroundings. The following paragraphs will give a glimpse of these potential applications for three common brain disorder groups: neurodegenerative diseases—with an emphasis on Parkinson’s disease (PD)—multiple sclerosis, and brain tumors.

#### Neurodegenerative diseases

Considering the aging population, it will be inevitable that—with the current insight and treatment options—neurodegenerative diseases will become one of the main diseases to be dealt with in the near future. For most neurodegenerative disorders, the diagnosis is made clinically, based on the presence of specific symptoms (such as bradykinesia combined with rigidity and/or tremor in PD; [58]). However, there is a strong need for adequate biomarkers. For instance, at the time of diagnosis, more than 50% of nigrostriatal dopamine neurons have already been lost in PD [59]. Given that PD likely starts up to 20 years before the diagnosis, the detection of these preclinical stages is especially relevant for the development of neuroprotective treatments [60]. Furthermore, at the time of diagnosis, it is not always easy to clinically separate different neurodegenerative disorders, such as PD and atypical parkinsonism. Since these different neurodegenerative disorders have different disease trajectories, good biomarkers can help in prognosis. Finally, post-mortem studies have made clear that the pattern of neurodegeneration differs markedly between patients with the same disorder. For example, the integrity of brain stem nuclei differs between PD patients with a tremor-dominant or non-tremor clinical phenotype [61]. This has led to a search for MRI biomarkers to identify individuals in the preclinical phase [62], and to differentiate between neurodegenerative disorders or disease subtypes [63]. In recent years, several structural MRI sequences enabled researchers to image the integrity of small brain stem nuclei, such as the locus coeruleus (LC) and the substantia nigra (SN), for example, using neuromelanin-sensitive imaging, iron mapping, and free water mapping [64–66]. For small regions like the LC, which has the shape and size of a spaghetti noodle, ultra-high-field imaging at 7 T has recently shown that in PD specific subregions are affected (i.e., the caudal more than the rostral portion of the LC) [67]). Furthermore, 7 T is able to provide a much more fine-grained view of neurodegeneration in specific subregions of the substantia nigra in PD, allowing, e.g., nigrosome imaging [68, 69]. Such detailed insights are necessary when making inferences about the pathophysiology of PD, such as how pathology spreads through the brain. More specifically, in PD there is recent evidence for a “body-first” versus a “brain-first” initiation of alpha-synucleinopathy, which differs between patients, and which then spreads in a prion-like manner through the rest of the nervous system [70]. Discerning these patterns in vivo with MRI requires a very high spatial resolution in the order of micrometers, something that is currently only achievable ex vivo using postmortem samples, with acquisition times approximating 15 h, even at 7 T [71, 72]. At 14 T, this spatial resolution can be achieved in a much shorter acquisition time and will, therefore, be feasible for in vivo imaging, while one can simultaneously make use of the larger image contrast that comes with increasing field strength [73]. This could provide the necessary anatomical specificity to identify patterns of pathology in subregions of the different brain stem nuclei (e.g., substantia nigra, raphe nuclei, and locus coeruleus), shedding light on the importance of these nuclei from a diagnostic perspective as well as their interaction and role in the early stages of PD. Another interesting player that in recent years has increasingly been recognized as both causal and sustaining factor in neurodegenerative diseases is the immune system [74]. For instance, in PD, evidence points to immune cells and signaling molecules potentially causing or at least facilitating neurodegeneration in the substantia nigra [75]. Elucidating the role of the immune system in PD and other neurodegenerative disorders may provide a new, not previously explored path towards better treatment [74]. Current insights into imaging the immune system will be discussed in the next paragraph.

#### Neuro-oncology

Tumours in the brain—whether primary or metastatic—are difficult to treat due to a variety of factors, including their location often within eloquent areas of brain tissue, the presence of the blood–brain barrier, and the sensitivity of the brain to radiation. Consequently, they have a detrimental effect on a patient's quality of life and are generally associated with a high mortality rate. In recent years, preclinical evidence has emerged that the microenvironment surrounding the tumour cells—the TME (tumour microenvironment) or metastatic niche in case of metastases—plays a crucial role in progression and survival of these cells [76, 77]. This microenvironment is formed through tumor-derived factors and extracellular vesicles, ultimately leading to alterations to the normal brain microenvironment and recruitment of tumor-associated immune cells including tumor-associated macrophages (TAMs). Due to the emergence of the tumor microenvironment, we now have two targets to aim at: the tumor cells themselves, and the microscopic environment surrounding these cells. Previous studies at 7 T have shown the added value of ultra-high-field (7 T) MRI in characterizing and delineating brain tumors on both structural and metabolic level using high-resolution structural imaging and metabolic imaging techniques, such as MR spectroscopy and chemical exchange saturation transfer (CEST), and it can be expected that 14 T will provide even more potential metabolic biomarkers due to its much increased spatial and spectral resolution [78–81]. However, the tumor microenvironment—and in particular the tumor-associated immune cells, such as TAMs—provides an additional and perhaps even more important imaging target with the advent of immunotherapy as a game changer in oncology [82, 83]. Considering the small scale on which the immune system operates, current imaging studies mainly use radioactive PET and SPECT isotopes to label immune cells or target immune cell receptors or substances, such as cytokines, while MRI is generally restricted to indirect imaging of subtle blood–brain barrier (BBB) leakage as a proxy of immune-mediated increased BBB permeability due to the limited specificity of most MRI sequences [84, 85]. To improve specificity, ultra-small paramagnetic iron oxide particles (USPIOs) such as Ferumoxytol are now actively studied both as a contrast agent leaking out of the vessels through a leaky BBB, as well as a molecule that is taken up by macrophages that subsequently traffic through the BBB to the tumor site, i.e., a ‘direct’ visualization of the immune system [86]. USPIOs (and the phagocytic cells in which they are taken up) can be visualized with MRI due to their magnetic susceptibility, which increases with magnetic field strength and provides two opportunities for 14 T MRI: decreasing the dose and tracking these iron-labeled cells with unprecedented spatial and temporal resolution, thereby gaining insight into their role within the tumor microenvironment in vivo, something which has so far mainly been done in animal models [87]. Another method of tracking TAMs is by means of fluorine-19 (19F) MRI. 19F MRI makes use of perfluorocarbon-containing nanoparticles that—similar to USPIOs—are taken up by macrophages and can be tracked to sites of inflammation. A recent preclinical study showed the great potential of this technique for imaging the intricate tumor microenvironment [88]. Current ultra-high-field strengths of 7 T are not sensitive enough to detect the small signal from 19F, necessitating unrealistically high doses and, therefore, restricting its use to preclinical studies. 14 T MRI, however, would have the necessary SNR to detect 19F in vivo, providing another way of visualizing the tumor microenvironment and its role in tumorigenesis. In summary, 14 T MRI could be able to probe the tumor microenvironment within the brain on a microscopic scale, both with its ultrahigh spatial resolution and its sensitivity to molecules and molecular pathways, elucidating how this microenvironment is sustained or—in case of metastases—*created*, opening up novel opportunities for treatment and perhaps even prevention of tumorigenesis.

#### Multiple sclerosis

Multiple sclerosis (MS) is a neuroinflammatory and neurodegenerative disease of the central nervous system [89], occurring in 2.8 million people worldwide. MS is based on focal demyelination, which causes lesional pathology in the white and grey matter. On conventional MR imaging, white matter pathology can be visualized relatively well, but in the grey matter this is highly problematic. Due to the lower myelinisation and different contrast in the cortex especially, most lesions remain invisible on sequences, such as T2-FLAIR [90]. However, it has been shown that cortical lesions are clinically highly relevant, with stronger correlations with disability and cognitive impairment than white matter lesions [91]. Recently advances have been made, using double inversion recovery (DIR) imaging, but this still only visualizes up to 20% of all cortical lesions [92]. Higher field strengths (7 T) have been used, doubling the yield compared to 3 T imaging, but detection rates are still not comparable to white matter lesions (> 90%) [93]. Therefore, current state-of-the-art neuroimaging is unable to adequately visualize cortical lesions in MS, while this is crucial to understand the pathophysiology of MS. Recent insights suggest that it is important to zoom in even further, suggesting layer-specific pathologies in MS, especially in layer 6, which is densely connected to the thalamus. Lesions in layer 6 may thereby cause large-scale network dysfunction [94]. Unfortunately, it is impossible to test this hypothesis with 3 T or even 7 T MRI. 14 T applications would allow us to develop methods that can further enhance in-vivo visualization of cortical lesions in MS.

### Medical applications

In vivo visualization of anatomy and physiology of the brain has often been the first application whenever big steps in magnetic field strengths were made in MR systems, with examinations of other parts of the body rapidly following. With whole body MR systems, all organs and tissues of the human body can be studied in a non-invasive way, giving in vivo access to morphology, physiology, and metabolism in the development of many different diseases. However, technical issues that are present for 14 T imaging of the head, become even more prominent when imaging the body. With larger field of views to capture the body circumference, and with greater differences in magnetic susceptibility between and within tissues, homogenizing *B*_1_ and *B*_0_ is even more challenging. The use of additional external shim coils for static *B*_0_ shimming [30], and the proper mitigation of inhomogeneous RF fields with, e.g., the TIAMO approach [95] with rapid *B*_1_ estimation [96] is indispensable. If the increased sensitivity is used to increase the spatial resolution of 1H imaging, then proper mitigation of motion is a must.

Where the previous section dealt with neurological and psychiatric disorders primarily involving the brain, here we outline how ultra-high-field imaging can improve our insight in diseases that affect multiple organs, for example auto-immune disorders and cancer. With nearly 4% of the world's population affected by one of more than 80 different autoimmune diseases and about 1 in 6 deaths related to cancer (WHO), these diseases put an enormous burden on modern society. New insights obtained with 14 T imaging will provide new fundamental knowledge necessary for developing improved detection, prevention, and treatment strategies. Nowadays, personal genetic predispositions or mutations in samples from abnormal tissues are used to characterize (risk of) disease, assessing its stage and predicting prognoses and response to therapy. Clinical data are often obtained from physical examinations and detailed ex vivo analyses of tissue or blood samples. However, collecting representative samples can be challenging, and heterogeneities across tissue or (different) organs in time or location are difficult to retrieve. MR imaging in particular, allows (repeated) non-invasive in vivo assessment of intact tissue status as well as insight in many dynamic processes. 14 T examinations will not become mainstream clinical evaluations, but in selected patient groups undergoing specific treatments the effect of these treatments can be studied in vivo.

A 14 T whole body MRI system can provide detailed maps of many different morphological and functional contrasts with an unprecedented spatial resolution, improving characterization of lesions without the current difficulties of spatially matching in vivo data to microscopy of excised specimens. Moreover, metabolic changes preceding functional and anatomical anomalies in disease development can be studied on a mesoscopic scale. Recent work with advanced spectroscopy methods resolved and identified approximately 29 metabolites at 15.2 T in an in vivo mouse brain [97]. Probing metabolic pools not only with proton spectroscopy but also with MRI of other nuclei facilitates a new window on metabolism, which can lead to an in vivo visibility of function and chemistry that has not been reached before, when extrapolating possibilities in the brain described at 7 and 9.4 T [98]. The sensitivity of 14 T will help visualize heterogeneity in chemical processes in the micro-environment of cancer, inflammation, or their interplay. It allows characterization of blood (micro-)vessels (walls) to the level of regeneration and visualization and characterization of immune responses in the lymphatic system. Cell mobility in organs can be studied by (single) cell tracking.

When taking a closer look at more specific methodology to be used at 14 T, many different opportunities arise. Chemical exchange saturation transfer (CEST) MRI utilizes the chemical exchange of protons between a solute molecule pool and the water pool for indirect detection of the solute pool. By prolonged but relatively low-power RF irradiation at the solute pool resonance frequency, indirect detection is possible by observing corresponding changes in the water pool magnetization [99–101]. Due to the high water-pool signal the solute molecule can be detected with significantly enhanced sensitivity by this indirect approach, which enables the generation of highly resolved metabolic maps. The spectral separation of different metabolites in the solute molecule pool increases linearly with field strength, allowing for the separation of creatine-associated guanidine signals at 2 ppm, phosphocreatine-associated signals at 2.7 ppm, the amide proton signals at 3.5 ppm, as well as rNOE associated with phosphocholines [102] and substrate binding [103] at − 1.8 ppm. Peak separation is also crucial for ratiometric approaches that can measure the pH from CEST effects [104], enabling the non-invasive, high-resolution 3D mapping of pH in vivo.

Different metabolites can also be measured directly, as they contain protons or other X-nuclei that possess nuclear spin. Two hallmarks of cancer, an altered cell proliferation and an altered energy metabolism, cause metabolic changes accompanying the important malignant transformation of primary cancer into metastasized disease. In vivo insight in the MR–visible molecules of the phospholipid metabolism of solid malignancies in, e.g., the prostate [105, 106] or the breast [107] at 7 T needs improved localization in smaller voxels to be related to the first appearance of metastases of these tumors. However, non-invasive detection of metastasized lymph nodes with imaging is one of the biggest current challenges in radiology, surgery, and oncology [108]. The use of ultrasmall superparamagnetic iron oxide (USPIO) nanoparticles at 7 T as an aid in the detection of nodal metastatic disease [109, 110], has great potential for sub-nodal detection of metastases at 14 T, narrowing the gap between histopathologically detected micro-metastatic disease in removed nodes and in vivo detection possibilities. Combining USPIO-enhanced imaging of relevant lymph nodes for prostate and breast cancer with 31P metabolic imaging and spectroscopy of the primary tumor provides detailed insight in the changes in phospholipid metabolism at the onset of metastatic spread of these tumors.

With 14 T multi-nuclei (31P, 1H) (spectroscopic) imaging not only characterization of the metabolic state of cancer tissue is possible with unprecedented sensitivity and spatial resolution, it will allow to dynamically monitor tumor metabolism and pH of tumors and their microenvironment in vivo in patients under treatment [111]. Knowledge of in vivo tumour metabolism and metabolic response in the tumour microenvironment will aid the development of new chemotherapeutics and drug delivery systems as it offers vital feedback on what actually happens in vivo. A breakthrough in understanding drug resistance in cancer can be achieved by dynamically monitoring metabolism of the tumors during treatment, or simply by observing number, size and perhaps texture of nearby lymph nodes [112], keeping the microenvironment and immune response intact. When related to drug efficacy and mRNA sequencing for each individual subject, variability in treatment efficacies between subjects can be better understood.

In the field of cell therapies, whether immune cell therapy against cancer or stem cells in for example, stroke or cardiac failure, there is a strong demand for imaging the course of these therapies. The field is growing at an explosive rate and the few FDA-approved therapies are extremely expensive, and outcomes are highly variable at the individual patient level. There is currently no method to understand, particularly at an early stage, whether a specific patient will respond or not, and thus the overall success rate for cell therapy trials is around 30–40%. A noninvasive technique to assess cell numbers and location in different regions of the body of patients under treatment, can help the assessment of treatment, preferably in a longitudinal manner. Imaging of the therapeutic cells would be a perfect solution. This would allow assessment of where the cells are, their numbers and possibly their functionality at relevant sites in the patient. This approach needs the highest MRI sensitivity possible. For quantification cell labeling with ^19^F MRI may be the best candidate, because this technology is noninvasive, does not use ionizing radiation or radioactive tracers (and so is not restricted by half-life decay), it is quantitative (for calculation of cell numbers, [113]), and stable [114]. However, sensitivity is the main limiting factor, which would experience a big gain when moving to 14 T.

Yet, another application of ultra-high-field MRI would be to longitudinally study metabolic rewiring of innate immunity. Innate immune rewiring—as a result of infection, vaccination or atherothrombotic events, such as myocardial infarction (MI)—elicits a strong immune response driven by activation of the hematopoietic system [115, 116]. It induces proliferation of hematopoietic stem and progenitor cells (HSPCs) in the bone marrow and subsequent HSPCs seeding in the spleen. Differentiation of activated HSPCs and the ensuing massive production of particularly inflammatory monocytes, which mainly rely on anaerobic glycolysis, is responsible for the significant increase in glucose consumption and overall metabolic activity observed in the bone marrow. This profound metabolic rewiring ultimately orchestrates the proliferation, egress of leukocytes from hematopoietic organs (‘leukocyte dynamics’) and drives inflammatory and reparative immune responses. Proton spectroscopy at 14 T can identify the size of the glucose pool in the spectrum downfield of water in the spleen, next to a ‘normal’ metabolic fingerprint of the same tissue. Using deuterium metabolic imaging (DMI) [117], or 13C-labeled glucose as a tracer, the metabolic fate of small amounts of glucose in the spleen and/or in bone marrow can be followed, identifying the level and preferred pathway of energy consumption in these tissue. Glucose metabolism can be mapped longitudinally in subjects who were BCG or SARS-CoV-2 vaccinated, who recently recovered from COVID-19 or from an atherothrombotic event, such as myocardial infarction and stroke, and linked to extensive metabolomics analyses of peripheral blood mononuclear and progenitor cells (from the bone marrow and spleen).

These examples are only a handful of opportunities that arise in more fundamental understanding of (dynamic) processes in different diseases once our patients can be safely examined in a 14 T MR system.

## Concluding remarks

The application of established HTS technology to construct a whole-body 14 T system will ultimately open new possibilities for research in multiple fields of MRI/MRS. The construction and testing of the system will take a number of years. It will also be necessary to perform rigorous testing before human subjects and patients can be scanned in the system. This will require both a comprehensive programme of local experiments, and extensive collaboration within the scientific community. We hope to become the first of multiple 14 T sites, and that 14 T will become an established field strength for research. By basing our approach on collaboration and open science we hope to support the development of 14 T sites worldwide and are happy to report that in February 2023 the Dutch Organisation for Scientific Research (NWO) awarded a large-scale research infrastructure grant to the DYNAMIC consortium to develop the system proposed here.

## Data Availability

No data are presented (it is a review) so there is no need for a data availability statement.
